# Destructive and Non-Destructive Evaluation of Fibre-Reinforced Concrete: A Comprehensive Study of Mechanical Properties

**DOI:** 10.3390/ma15134432

**Published:** 2022-06-23

**Authors:** Hadee Mohammed Najm, Ominda Nanayakkara, Mohanad Muayad Sabri Sabri

**Affiliations:** 1 Department of Civil Engineering, Zakir Husain Engineering College, Aligarh Muslim University, Aligarh 202002, India; gk4071@myamu.ac.in; 2 Department of Civil Engineering, Xi’an Jiaotong-Liverpool University, Suzhou 215000, China; 3 Peter the Great St. Petersburg Polytechnic University, St. Petersburg 195251, Russia; mohanad.m.sabri@gmail.com

**Keywords:** non-destructive test, ultrasonic pulse velocity (UPV), modulus of elasticity, fibre-reinforced concrete (FRC), Poisson’s ratio

## Abstract

Ultrasonic pulse velocity (UPV) and rebound hammer tests are accepted as alternatives to destructive testing to determine the compressive strength, dynamic modulus of elasticity, and Poisson’s ratio, which are needed for structural design. Although much work has been conducted for plain concrete, the research data for fibre-reinforced concrete (FRC) is insufficient. In this regard, this study explains the correlations between compressive strength, rebound hammer, and UPV tests for plain concrete and FRC contains 0.25%, 0.50%, and 1.00% of 30 mm and 50 mm long steel fibres. A total of 78 concrete cube and beam specimens were tested by direct, semi-direct, and indirect UPV and rebound hammer test methods. The study found that the rebound hammer test is more suitable for measuring the compressive strength of matured FRC than young concrete. The UPV test revealed that the volume fraction does not, but the length of steel fibres does affect the UPV results by the direct test method. The UPV direct method has the highest velocity, approximately two times the indirect velocity in FRC. UPV measurements can be effectively used to determine the dynamic modulus of elasticity and Poisson’s ratio of FRC. The dynamic elastic modulus increases while the Poisson’s ratio decreases for the same steel fibre length when at increasing FRC fibre content. The results of this study will be significant for non-destructive evaluations of FRC, while additional recommendations for future studies are presented at the end of the paper.

## 1. Introduction

The flexural and shear stiffness of concrete elements should be calculated to assess the lateral displacements and deflections. Steel fibres provide great advantages in high-rise concrete buildings and long-span concrete bridges [1]. The modulus of elasticity and the Poisson’s ratio are the most important mechanical properties to consider determining the flexural and shearing stiffness of concrete structures [2]. Vertical deflections and story drift at the service limit state directly relate to the elastic stiffness of the concrete elements. Poisson’s ratio is commonly assumed to be equal to 0.20 in most building codes for estimating the modulus of elasticity of plain concrete, (i.e., without fibres). According to Kumar et al. and Najm et al. [3,4,5] and fibres are used to improve toughness, ductility, and residual strength at the material level, yield capacity, energy absorption, and yield strength at the structural level. Steel fibres are now used in a wide range of construction projects, from high-performance concrete structures to ultra-high-performance concrete structures [6]. Parra-Montesinos [7] argued that high-performance fibre-reinforced concrete (FRC) is suitable for structural members where an adequate seismic behaviour is required.

The equations for estimating the elasticity modulus and Poisson’s ratio of FRC are not included in modern building codes, despite wider applications of steel FRC [8]. An in-depth understanding of FRC’s measurement methods is necessary because the elastic parameters data are essential for structural design [2]. Concrete’s dynamic and static elastic properties are commonly assessed using destructive and non-destructive tests. Using non-destructive testing saves time and money because the tests are quick and tested components are not damaged. Using non-destructive testing, structural defects, concrete’s elastic modulus, strength, the thickness of a concrete cover, as well as the evolution of concrete’s curing and hardening over time can all be determined. The ultrasonic pulse velocity (UPV) test is widely used to evaluate concrete structures because of its ease of use, versatility, and repeatability. According to Hassan and Jones [9], the UPV test is the most reliable, easy, and portable non-destructive method for evaluating materials’ elastic properties. UPV test results have been effectively used to investigate the properties of rubberized concrete [10,11], lightweight concrete [12,13], fly ash self-compacting concrete [14], concrete containing ground granulated blast furnace slag [15], concrete at high temperature [16], and concrete with frost influence [17]. The high sensitivity of the UPV method makes it possible to detect discontinuities in deep elements and extremely small discontinuities. In addition, the test can be carried out with only one side of the structural element available for testing. Blitz and Simpson [18] concluded that the UPV test equipment “must generate a pulse that can be transmitted to concrete via a transmission and a reception transducer, thus turning mechanical energy into new impulses of the same frequency”. It is possible to configure these emitter and receiver transducers in three different ways (direct, semi-direct, and indirect). Malhotra V. et al. [19] shows that, “The direct method is one of the most satisfactory methods since the maximum energy of the pulse is sent and received. The semi-direct method is satisfactory, but its configuration must be performed with greater caution to avoid losing the signal. The indirect method turns out to be the least satisfactory since the signal is relatively low”.

It has been observed through the literature that there are existing experimental works to investigate the mechanical properties of fibre-reinforced concrete. However, there is minimum available experimental work to evaluate the effect of length and volume fraction of steel fibre on the concrete compressive strength, modulus elasticity, and Poisson’s ratio, which is investigated using destructive and non-destructive tests. The results of this study can be a reference for future studies and provide wide data for fibre-reinforced concrete studies.

## 2. Research Significance

This paper aims to propose empirical relationships for estimating the compressive strength, modulus of elasticity, and Poisson’s ratio of concrete reinforced with steel fibres, using measured data of UPV tests. This research is also intended to evaluate the effect of test setup (direct, semi-direct, and indirect), and establish a relationship between the strength of FRC and the characteristics of steel fibres (length and fibre fraction). The experimental study shown in this paper includes non-destructive tests and destructive tests. The non-destructive properties were analyzed based on variables such as fibre length, fibre content, and test setup (direct, semi-direct, and indirect). The length of steel fibres was 30 mm and 50 mm. Fibre volume fractions in the concrete varied between 0, 0.25, 0.50 and 1.00%. The non-destructive tests used the ultrasonic pulse and rebound hammer tests which were performed on 15 specimens (beam type). The destructive were performed on the 65 cube specimens. The recorded pulse rates allowed for calculating the values of the modulus of elasticity and Poisson’s ratio.

## 3. Materials and Methods

### 3.1. Material Properties

#### 3.1.1. Cement

Portland cement (42.5N) was used in this research. Cement was obtained from a single supplier to ensure the quality of the material is the same for all concrete mixes.

#### 3.1.2. Aggregate

**Figure 1:**
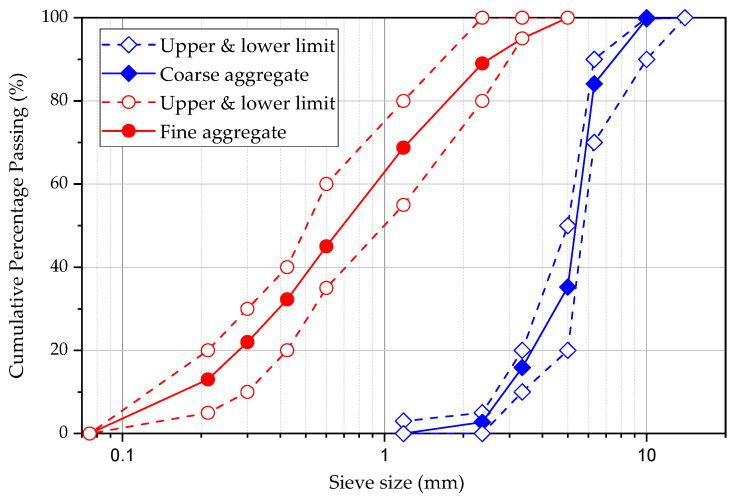
Sieve analysis data of coarse and fine aggregates.

The coarse and fine aggregate grading and characteristic are given in Table A1 (Appendix A). The maximum size of coarse aggregate is 15 mm, and the coarse type is crushed Basalt. The size of coarse aggregate was kept relatively small to increase the mixing efficiency of steel fibres within concrete. It is generally suggested that the fibre length should be at least twice the maximum size of the coarse aggregate for steel FRC. In this research, the lengths of steel fibres were used as 30 mm and 50 mm, which can satisfy the requirements. The sand is river sand with a slightly higher water absorption than coarse aggregates. The sieve analysis test results of coarse and fine aggregates are shown in Figure 1.

#### 3.1.3. Steel Fibres

Steel fibres were used in this investigation as steel fibres are the most widely used fibres in FRC. Steel fibre is very good at increasing the most of properties of concrete. The length of steel fibre used in this research is 30 mm and 50 mm. The steel fibre type and properties such as strength and modulus are shown in Table 1.

### 3.2. Mixture Proportions

**Figure 2:**
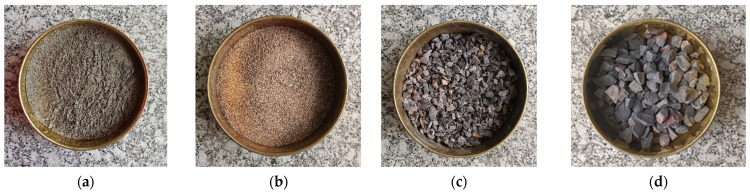
(**a**) Commercial cement; (**b**) river sand; (**c**) natural coarse aggregates (NCA)—10 mm; (**d**) natural coarse aggregates (NCA)—20 mm.

The final mixture proportion is shown in Table 2 & Figure 2, and the concrete mix design for each material is shown in Table A2 (Appendix A). Two different lengths of steel fibres (30 mm and 50 mm) have been used. Based on the total concrete volume, three fibre fractions of 0.25%, 0.50%, and 1.00% were added to the specimen. There are seven groups of concrete mixture proportions considering two different fibre lengths and three different fibre volumes, including the specimen without fibres. Steel fibres, at volume percentages of 0.25%, 0.50%, and 1.00%, will occupy some specimen space, and each mixture’s proportion will be accordingly designed.

#### 3.2.1. Mixing Procedure

The mixing procedure was based on ASTM manual (ASTM C 109, ASTM C 1074-04, ASTM C 1611). Concrete was mixed in a drum mixer which can mix up to 100 kg of concrete each time. Coarse and fine aggregates were first to dry and mixed for one minute, and then cement was mixed with aggregates for another minute. Thirdly, water was added and mixed until a uniform mix was achieved. Fibre was added to the mix slowly and uniformly, and the concrete was then mixed for five minutes. The water to cement ratio is relatively high, and therefore, admixtures were not added to the concrete mix.

#### 3.2.2. Dimensions of Specimens

The experimental work of the present study consists of 63 cubes and 15 beams with different dimensions, as shown in Table 3. A total of 78 specimens were cast to investigate the mechanical properties of concrete reinforced by different lengths (30 mm and 50 mm) of steel fibre to find its optimal use. For this purpose, 63 cubes and 15 beams were cast with varying percentages of fibre (0%, 0.25%, 0.50% and 1.00%) and used for UPV and compressive strength tests. Beam specimens, 1 to 4, have a fibre content of 0% and 0.25% 30 mm fibre in each group. The concrete mix for beam type 5 consists of one beam without fibre and six beams of 0.25%, 0.50%, and 1.00% fibre content with 30 mm and 50 mm fibre lengths.

### 3.3. Test Procedure

#### 3.3.1. Curing Procedure

According to the ASTM C192, the concrete specimens were kept in their moulds for 24 h after mixing and then cured in water at a constant temperature of 20 °C until test. The test age of the specimen is 3, 7, and 28 days and the specimens were taken out of water for UPV testing for less than an hour during the curing days. The cubes were tested for compressive strength at 3, 7, and 28 days. The beam specimens were tested for the UPV measurements at 3, 7, and 28 days.

#### 3.3.2. Compression Test

The test age of compressive strength is 3, 7, and 28 days for all specimens (ASTM C39). The compression tests were subdivided into four major groups, i.e., cube specimens with no fibre, cube specimens contained 0.25% 30/50 mm fibre, cube specimens contained 0.50% 30/50 mm fibre, and cube specimens contained 1.00% 30/50 mm fibre. To decrease the error of the test, three cubes for each group were tested.

#### 3.3.3. Rebound Hammer Test

**Figure 3:**
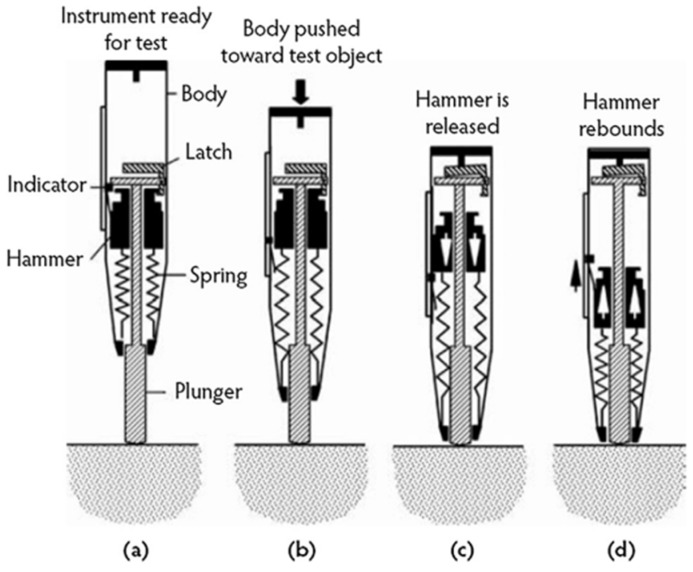
Operation of the rebound hammer.

The rebound hammer test is non-destructive testing that can measure the strength of finished concrete structures. The rebound hammer equipment is easy to carry, and the test procedure is simple. The rebound hammer can only estimate the compressive strength on the surface of concrete, and it is not very accurate in predicting the actual compressive strength of concrete. The rebound hammer test is also used to correlate the compression strength for UPV measurements (Figure 3).

This method can only determine the relative strength of concrete, which is based on the hardness of the specimen at its surface. If the concrete is exposed to the atmosphere for more than three months, the results may overestimate as much as 50% because of carbonation at the concrete surface. In this research, the rebound hammer test was conducted at 3, 7, and 28 days. Table 4 shows the standard for determining the quality of concrete by rebound hammer results and it can be used to determine the level of concrete used in this research. A smooth and dry surface of the concrete at the test point can promise the accuracy of rebound reading.

#### 3.3.4. Ultrasonic Pulse Velocity (UPV)

The UPV technique is one of the widely used and convenient methods that can provide test results at the lowest cost and rapid measurements. The UPV can determine the quality of concrete, as shown in Table 5.

The UPV is constant during the travel through the specimen unless the lateral dimension is too small and less than a certain minimum value. If the path is below a certain minimum value, the pulse velocity may be decreased, and the data may not be reliable. The ratio of the wavelength and the path dimension of the specimen relate to the reduction of velocity. Based on Table 6, the relationship between the frequency and the minimum dimensions of the travel path, 54 kHz transducer frequency was used. Additionally, the Pundit Lab (manufacturer) recommends that the path length be more than 100 mm when the maximum aggregate size is 20 mm.

Several factors can affect the compressive strength of concrete and some of those factors also influence the UPV. The relationship between these variables and the UPV will be investigated in this study. These variables are:

1. Age of concrete (3, 7, and 28 days).
2. Testing procedure (direct, semi-direct, and indirect UPV test).
3. Fibre percentage (0.25%, 0.50%, and 1.00%).
4. Fibre length (30 mm and 50 mm).

- Direct UPV measurements.

**Figure 4:**
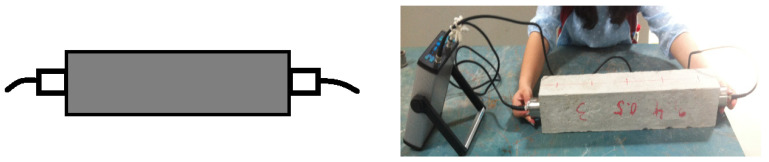
Direct UPV measurement.

The direct tests were conducted at the long side of each beam specimen as shown in Figure 4. The direct path length for these measurements was through the longitudinal direction of beams which are of 100 mm, 200 mm, 300 mm, 400 mm, and 500 mm long.

B. Semi-direct UPV measurements

**Figure 5:**
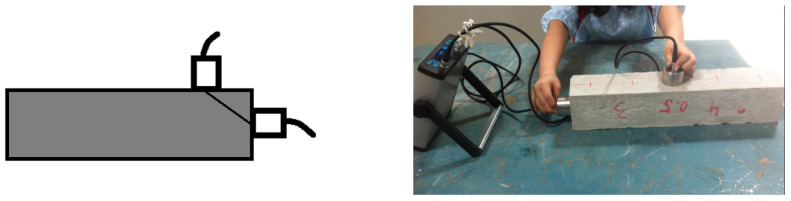
Semi-direct UPV measurement.

Generally, the accuracy of semi-direct UPV measurements is less than the direct measurements. Still, the concrete members such as walls and columns can only use semi-direct or indirect UPV measurements due to the limitations in transducer placement. The path length between transducers was calculated using the Pythagorean theorem. The transducer in the length direction was placed in 100 mm spacing along the beam, as shown in Figure 5. The UPV by the semi-direct method has four path lengths for each specimen at 158 mm, 255 mm, 354 mm, and 453 mm. This is the calculated distance between transducers in the diagonal direction.

C. Indirect UPV measurements

**Figure 6:**
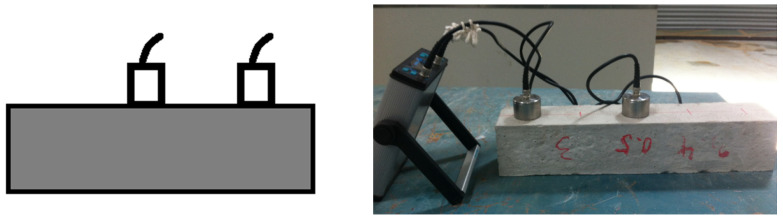
Indirect UPV measurement.

Although the indirect UPV measurements are poor sensitivity compared to the direct test, it is necessary to find the relationship between the UPV and the concrete properties by indirect UPV test because sometimes it is not possible to use the direct UPV measurements under some conditions. Both transducers are placed on the same surface of the FRC in this method, as shown in Figure 6.

**Figure 7:**
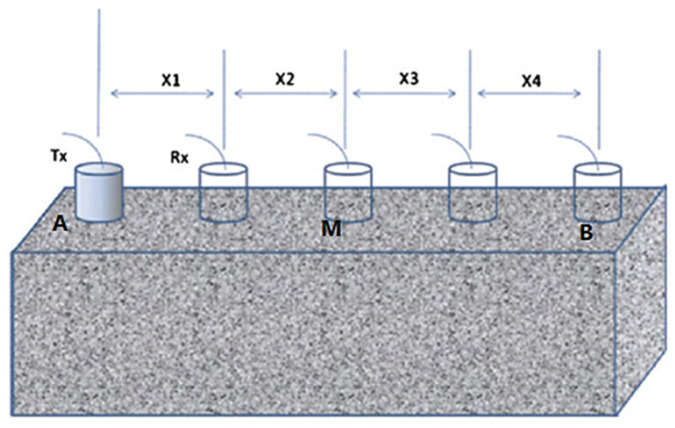
Schematic view of Indirect UPV test.

In this method, one transducer (Tx) was placed at position A and the other transducer (Rx) was placed at a distance of X1 away from position A, as shown in Figure 7. Then, change the location of the transducer Tx by a distance of X1 away from position A to position B. The average UPV and transmit time can be obtained from the path length of 200 mm by one transducer (Tx) is placed at position A and the other transducer (Rx) is placed at position M. Then, change the transducers to different locations so that the spacing between two transducers will be 200 mm. Theoretically, the UPV should be the same, and the transmit time will increase as proportional to the spacing between two transducers.

## 4. Results and Discussion

### 4.1. Compressive Test

**Figure 8:**
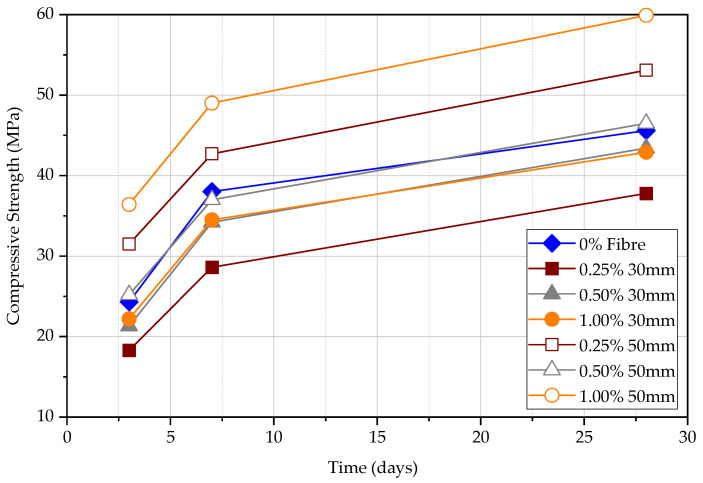
Compressive strength for different fibre content at 3, 7, and 28 days.

Compressive strength was measured at 3, 7, and 28 days as shown in Figure 8. The compressive strength of FRC will increase with the time from 3 days to 28 days, which agrees with existing knowledge. The specimen, which contains 0.25% of 30 mm fibre, shows the lowest strength in 3, 7, and 28 days compared to all other specimens. The compressive strength of concrete with 50 mm fibre is higher than that of the 30 mm fibre [20]. As the fibre content increases, the compressive strength of the concrete increases due to the effect of confinement although fibres are assumed to be effective only in increasing the toughness of concrete. As a result, steel fibres can significantly increase concrete capacity for tension and flexure; however, the increase in compression capacity is comparatively lower.

### 4.2. Rebound Hammer Test

**Figure 9:**
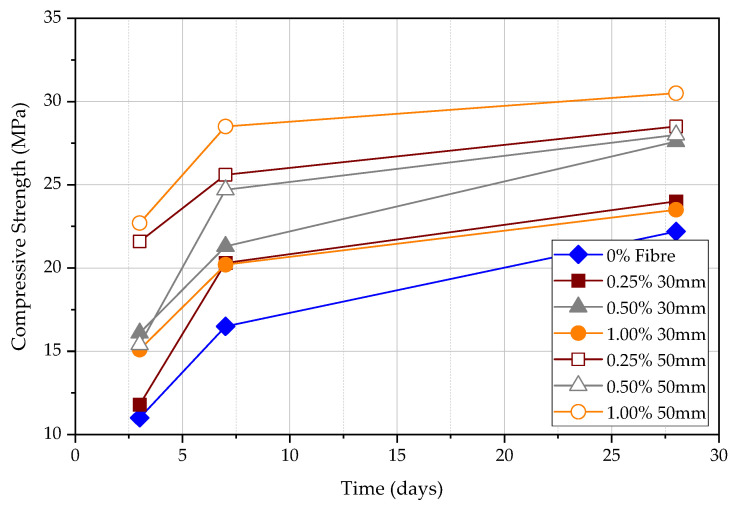
Compressive strength (MPa) using rebound hammer test result.

After calculating the average of the six readings of each type of specimen on a particular test day, the rebound hammer test result is shown in Figure 9. It can be seen that the strength of concrete, which contains 30 mm fibre is smaller than the strength of concrete which contains 50 mm fibre. This relationship between the rebound hammer test and the compressive strength test is consistent with the experimental results. Figure 9 shows that the strength of concrete without fibres has the lowest strength at 3, 7 and 28 days in comparison to FRC.

**Figure 10:**
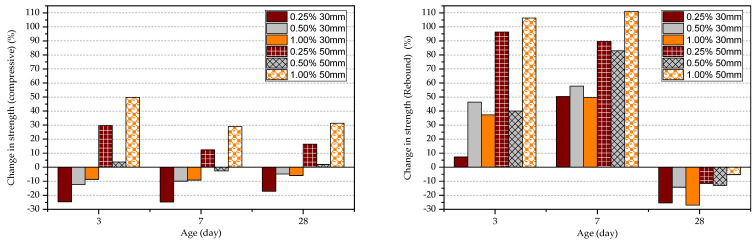
Percentage change in strength.

The percentage change in strength relative to the plain concrete was calculated in each FRC specimen as shown in Figure 10. The change in the compressive strength results is relatively small in comparison to the results of the rebound hammer test at the age of 3 and 7 days; however, the variation is similar at the age of 28 days. The compressive strength of normal Portland cement concrete reaches 90% at the age of 28 days relative to the strength in long term. The observation could be attributed to the method of measurement in each technique where direct compressive strength considers the effect of the full specimen while the rebound hammer test considers only the surface. In long term, the effect of fibre on the rebound hammer result is minimum while in short term the effect is significant. This could be because adjacent fibres can resist the rebound hammer pressure although the concrete medium is weak. This observation can confirm that the rebound hammer test is not suitable to obtain compressive strength data in FRC at an early age.

### 4.3. Ultrasonic Pulse Velocity (UPV) Test

#### 4.3.1. Direct UPV Measurements

**Figure 11:**
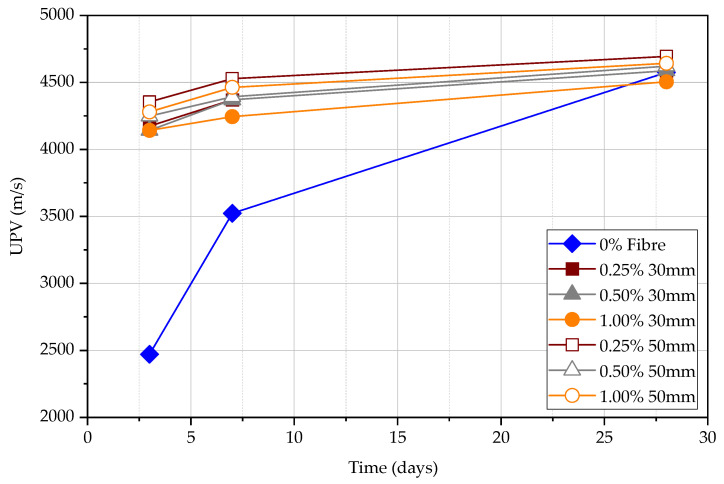
Direct UPV measurements at 3, 7, and 28 days.

**Figure 12:**
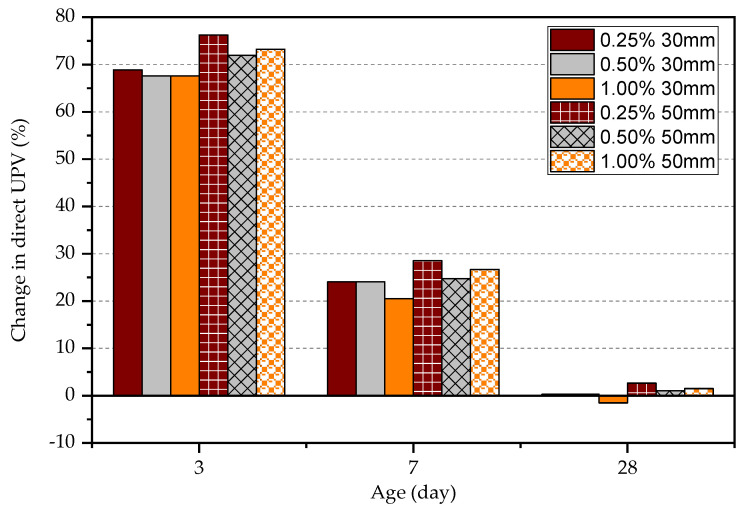
Change in direct UPV in FRC relative to plain concrete.

It is stated that when the compressive strength increases, the UPV increases. The UPV result follows approximately the same pattern for each kind of FRC at a given test age as shown in Figure 11. However, UPV measured in plain concrete shows that the difference between UPV in comparison to FRC is significant as shown in Figure 12. The difference in UPV between plain concrete and FRC is approximately 70%, 25%, and 0% for concrete at the age of 3 days, 7 days, and 28 days, respectively. Steel fibres within the concrete matrix can increase the UPV of specimens, especially at an early age even at the low maturity of concrete. This is because pulse wave travels much faster within the steel medium than porous concrete medium as previous studies have shown. The volume fraction of steel fibres seems ineffective in increasing the UPV; however, longer steel fibres can slightly increase the UPV measurements. The UPV in specimens with 50 mm long fibres is larger by 5.7%, 3.8%, and 2.0% at the age of 3, 7, and 28 days, respectively, in comparison to the specimens with 30 mm long fibres. Although the plain concrete matrix is weak, the FRC composite shows a higher UPV at an early age. However, matured concrete shows that the fibre content does not affect the measured UPV. In general, the UPV results of FRC at all ages exceed 4000 m/s while it is more than 4500 m/s at 28 days, which declares that the quality of concrete is good.

**Figure 13:**
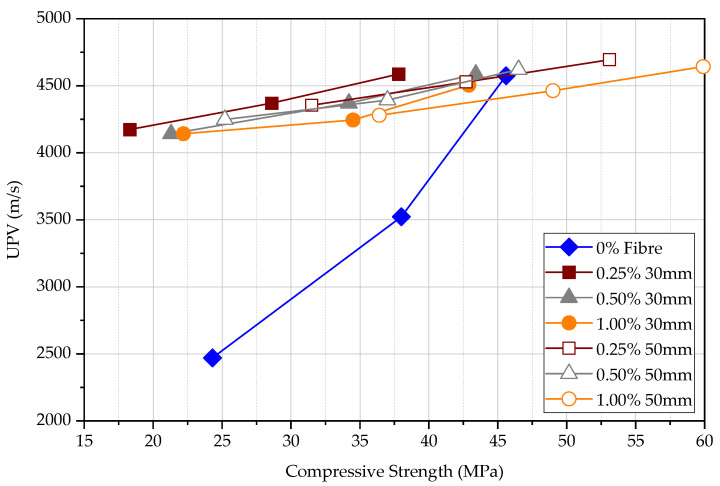
Direct UPV measurements vs. compression strength.

According to Figure 13, both the compressive strength and UPV increase with the age for all FRC and plain concrete specimens. For plain concrete, the UPV changes significantly with the compressive strength while the change in FRC is less significant. Therefore, if the UPV test is used to measure the FRC, it will give a relatively high estimation of compressive strength compared to the test for plain concrete. The influence is approximately two times at 3 days, 1.5 times at 7 days, and similar at 28 days. The compressive strength of FRC increases approximately at the same rate for all specimens. UPV is less influenced by fibres in comparison to the influence on the compressive strength, especially at the early age of concrete, as also explained according to Figure 12.

#### 4.3.2. Semi-Direct UPV Measurements

**Figure 14:**
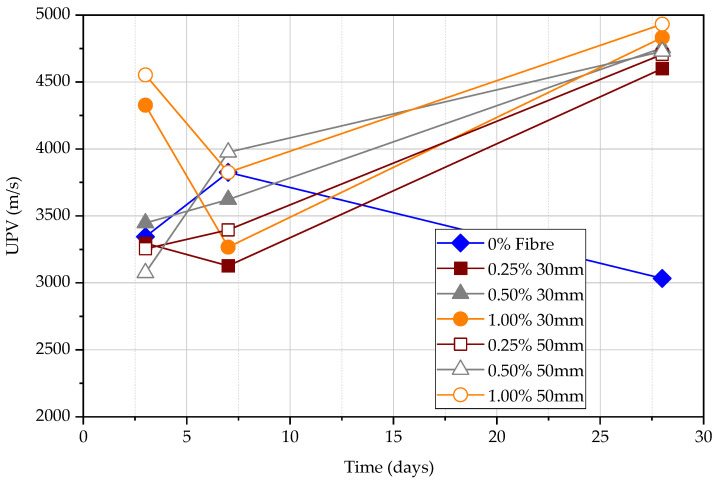
Semi-direct UPV measurements at 3, 7, and 28 days.

Figure 14 shows the semi-direct UPV measurements in all specimens at the age of 3, 7 and 28 days. The UPV in FRC and plain concrete at 28 days has a significant difference. There is no significant correlation between variables of fibre type, fibre content, and the length of the fibre. The most reasonable results of data are obtained at the age of 3 days and 28 days. The 1.00% 50 mm fibre content specimen shows the largest semi-direct UPV at 3 and 28 days. The lowest semi-direct UPV is the FRC specimen of 0.50% 50 mm fibre at 3 days. However, the smallest UPV occurs in the beam without fibre, and the largest UPV occurs in the beam with 1.00% 50 mm fibre. As there is no correlation between compressive strength and the UPV for the semi-direct test, the semi-direct test is less useful in determining the compressive strength. It is necessary to pay attention to the use of this method for concrete quality control.

**Figure 15:**
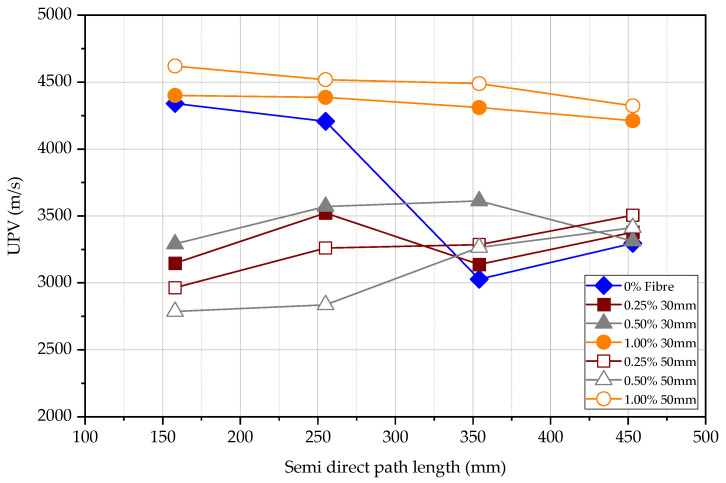
Semi-direct UPV measurement vs. path length at 3 days.

**Figure 16:**
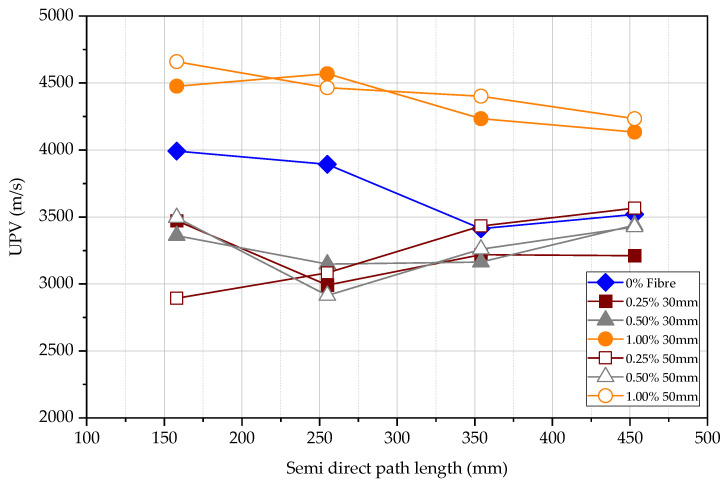
Semi-direct UPV measurement vs. path length at 7 days.

According to Figure 15 and Figure 16, the UPV of each type of FRC specimen is similar for different path lengths. Semi-direct UPV test data at 28 days of age is not available due to experimental misconduct. To increase the accuracy, an average UPV value could be used. However, the UPV decreases rapidly for the concrete specimen without steel fibre when measured path lengths increase. This may be attributed to the significant loss of signal due to the path length in the absence of steel fibres. Although the concrete with and without steel fibre could be named isotropic, the path length significantly influences the UPV of different types of concrete. Hence, the average semi-direct test data will be used for each kind of beam for further discussion and ignore data with a significant variation.

**Figure 17:**
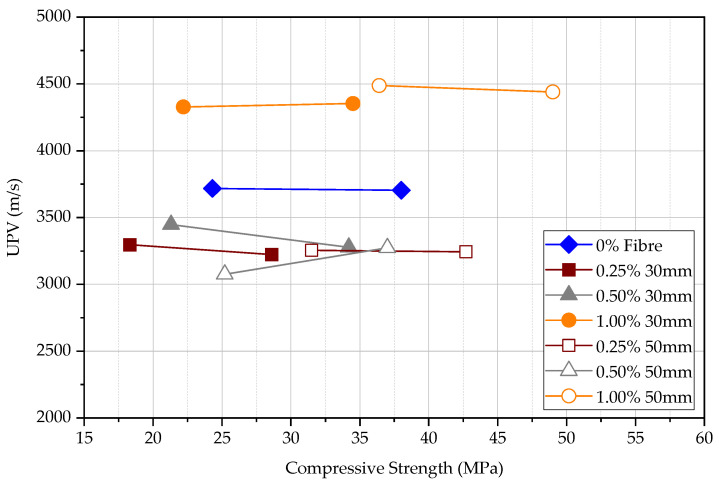
Semi-direct UPV measurements vs. compression strength.

The semi-direct UPV is approximately constant at different ages of concrete, although there is a significant difference in the measured compressive strength, as shown in Figure 17. For each specimen, the first data point represents the age of 3 days while the second data point represents the age of 7 days. Although the accuracy of a semi-direct test is low in FRC, the test method is more convenient than a direct test. Hence, to use the semi-direct method, it is very effective to gather a large number of UPV data of FRC and statistical analysis to make sure the results are reliable [21]. A strength test is often used for quality control of FRC, and many concrete tests often show a normal distribution pattern [22]. It is recommended to conduct more testing for concrete compressive strength and UPV, and then find the strength-UPV relationship for estimated strength by UPV. However, in this study, no certain relationships can be found because of little test data.

#### 4.3.3. Indirect UPV Measurements

**Figure 18:**
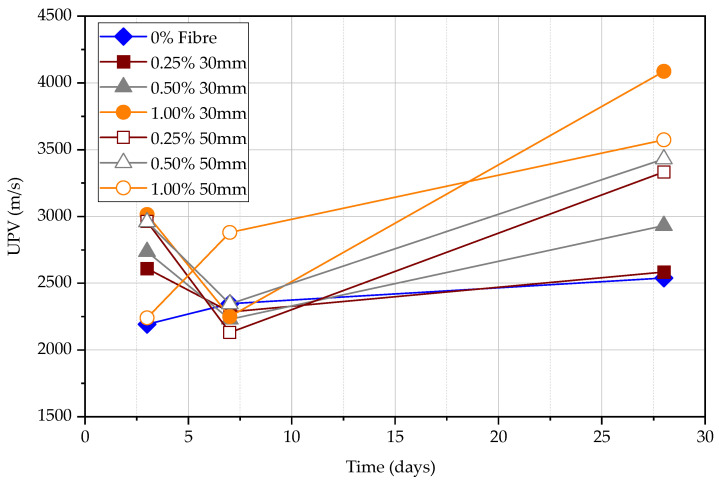
UPV indirect measurements with 100 mm path length.

**Figure 19:**
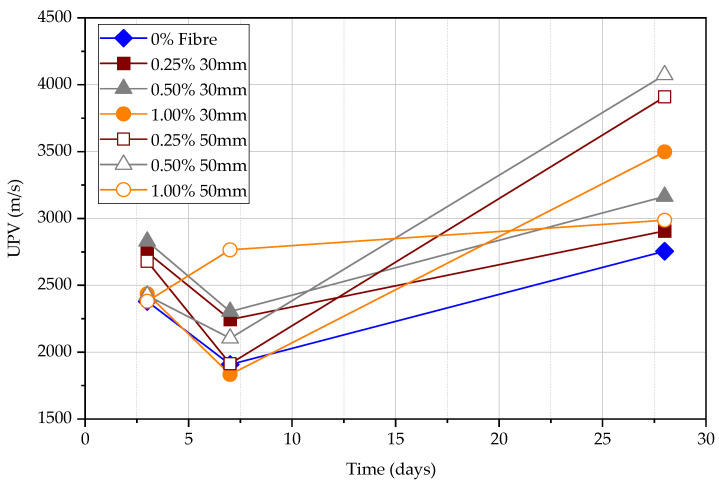
UPV indirect measurements with 200 mm path length.

Figure 18 and Figure 19 together with Table 7 shows indirect UPV measurements for all the specimens at 3, 7, and 28 days of age. It can be found that the UPV will increase for the different path lengths of the beam with increasing age. The UPV measured with different path lengths could be assumed to be constant for the same concrete specimen measured on the same day. However, results of different path lengths show that UPV on the same day is not well correlated with the amount of fibre content and the length. This error is partly due to the inconsistency of path distances between transducers which is difficult to accurately control. The beam length and path distance are relatively too small. The recommendation of the minimum path length is 100 mm. For the manual UPV test, it is possible that the actual test path length is less than 100 mm. Hence, the results are not reliable in this 100 mm path length indirect test stage. It is suggested that the data obtained from the longer path length of UPV measurements could be reliable as it can eliminate the effect of short path length. However, it should be at the same time noted that larger path length could affect data due to the non-homogeneous concrete materials and the loss of wave energy during its travel through concrete media. Table 8 shows that the difference between UPV values for a path length of 100 mm and 200 mm is less than 20%. This could provide some insight into the path length for the indirect test.

### 4.4. Comparative Relationships of Direct, Semi-Direct, and Indirect Measurements

Direct, semi-direct, and indirect UPV measurements are important measuring methods, and they are directly affecting the pulse velocity values. The average direct UPV is higher than the average semi-direct and indirect UPV on all days. The average semi-direct UPV is higher than the average indirect UPV on all days for all kinds of specimens. The surface velocity (indirect measurements) is about half of the direct velocity for the beam with and without steel fibres. This relationship can be used to determine the Poisson’s ratio and dynamic elastic modulus of concrete.

### 4.5. Poisson’s Ratio and Dynamic Elastic Modulus

**Figure 20:**
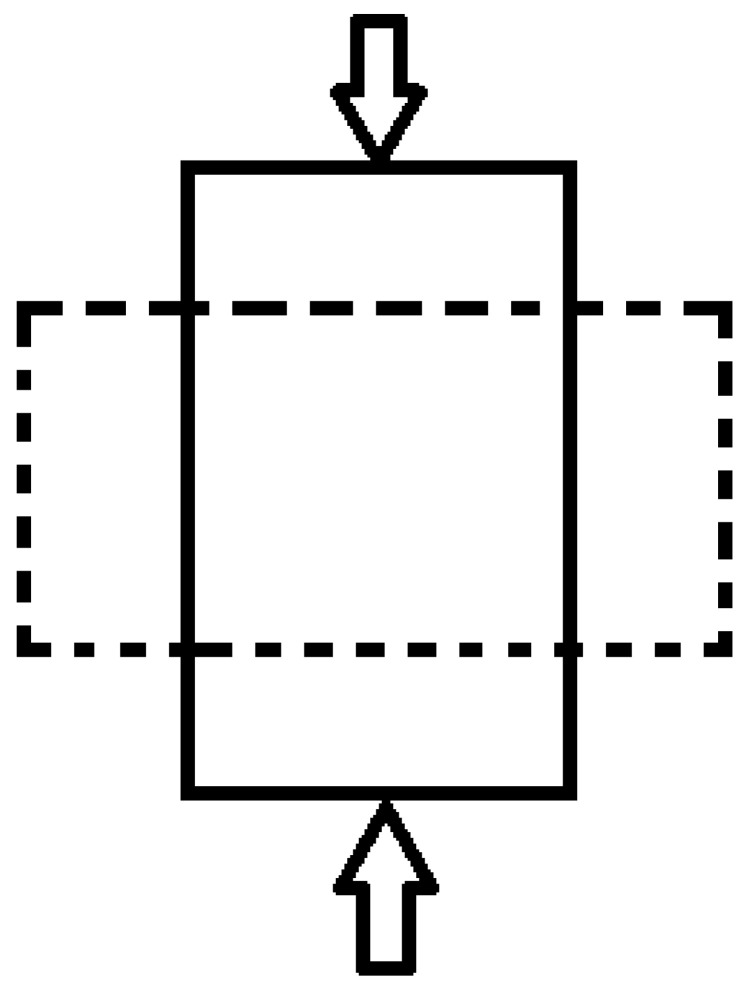
Length change of material under compression.

When a material is under compression, the length reduces in the longitudinal direction (axial), and the length increases in the lateral direction (transverse) as shown in Figure 20. The definition of Poisson’s ratio is the negative ratio of the relative axial strain to the strain in the direction of the applied force [23] (Equation (1)). Generally, including cementitious and concrete materials, the Poisson’s ratio for most materials varies between 0 and 0.5. Assuming the specimen is compressed along the axial direction, the Poisson’s ratio can be calculated as in Equation (1). For isotropic material, the relationship of shear modulus, Poisson’s ratio, and elasticity modulus can be shown in Equation (2).

(1) $$v=-\frac{d\varepsilon_{trans}}{d\varepsilon_{axial}}$$

(2) $$G=\frac{E}{2\left(1+v\right)}$$

where v is Poisson’ratio, $\varepsilon_{trans}$ is transverse strain, $\varepsilon_{axial}$ is axial strain, G is shear modulus (GPa), v is Poisson’s ratio, and E is elasticity modulus (GPa).

The dynamic elastic modulus ($E_{d}$) and Poisson ratio (v) can be calculated using the measured compression wave (P-wave) and shear wave (S-wave) velocity by direct measurement of UPV. Because of the multiple scattering in the concrete, it is difficult to determine the S-wave. Existing relationships to determine properties by wave velocity are presented as in Equations (3)–(5) [24]. Combining Equations (3) and (5), Equation (6) can be obtained.

(3) $$V_{p}=\sqrt{\frac{E_{d}\left(1-v\right)}{\rho \left(1+v\right)\left(1-2v\right)}}$$

(4) $$V_{s}=\sqrt{\frac{E_{d}}{2\rho \left(1+v\right)}}$$

(5) $$V_{r}=\frac{0.87+1.12v}{1+v}\sqrt{\frac{E_{d}}{2\rho \left(1+v\right)}}$$

(6) $$\frac{V_{p}}{V_{r}}=\frac{1+v}{0.87+1.12v}\sqrt{\frac{2\left(1-v\right)}{1-2v}}$$

where, $E_{d}$ is dynamic elastic modulus (GPa), ρ is density (kg/m^3^), $V_{p}$ is the compression (longitudinal) wave velocity (km/s), (*v*) Poisson’ ratio, $V_{s}$ is the shear wave (transversal) velocity (km/s), and $V_{r}$ is the surface (Rayleigh) wave velocity (km/s).

According to Equation (6), the dynamic Poisson’s ratio can be determined by measuring the UPV of compression and surface wave propagation. The UPV will change with the different properties of concrete such as its elastic stiffness and mechanical strength.

It has been reported that the correlation between the static modulus value and the dynamic modulus value determined by UPV is good and the error oscillates between 10–15%. The error for the resonant test is about 11–14%, indicating the UPV is more accurate [22]. Therefore, the UPV test can be used to determine Poisson’s ratio and modulus of elasticity of FRC. By the use of equations, the dynamic elastic modulus and Poisson ratio for different beam types without steel fibre and with fibres of different amounts and lengths can be easily obtained.

The density of 2500 kg/m^3^ was used for the FRC which is reasonable for normal aggregate concrete with steel fibres. In Table 9, results for the UPV tests of the beam without fibre are presented. It can be easily found that the modulus of elasticity and Poisson’s ratio increase with the age of the concrete. The modulus of elasticity is 15.14 GPa, 28.10 GPa, and 45.20 GPa at 3, 7, and 28 days, respectively. The Poisson’s ratio of normal concrete is between 0.100 and 0.200. According to the test results, the concrete Poisson’s ratio is between 0.062 and 0.229, which is consistent with the general concrete Poisson’s ratio.

**Figure 21:**
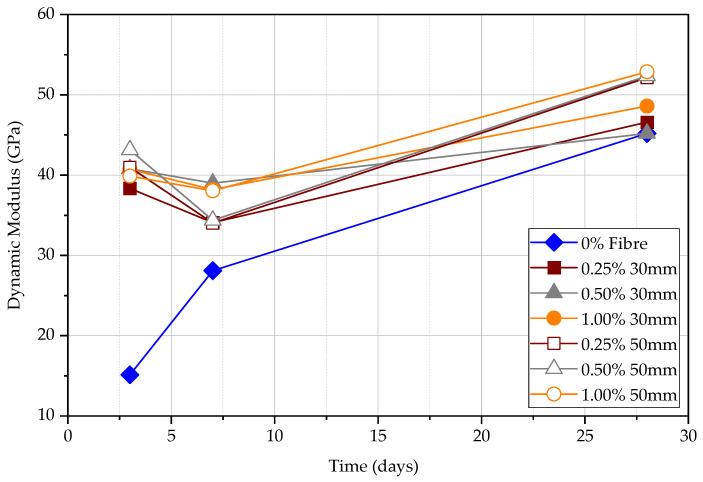
The modulus of elasticity (GPa) of beams at 3, 7, and 28 days.

**Figure 22:**
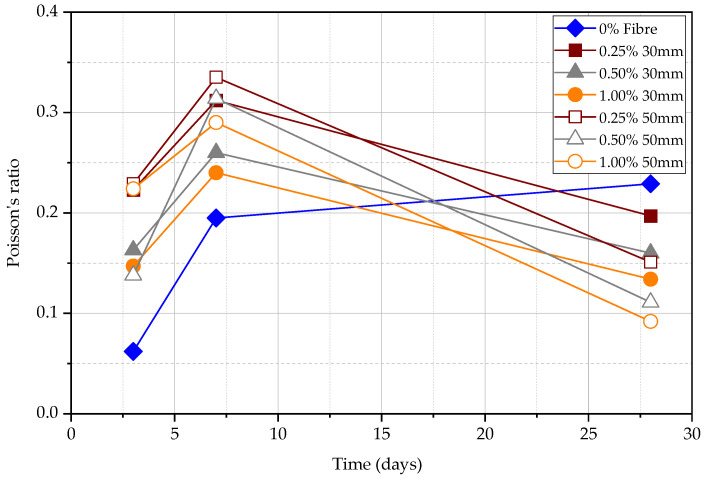
Poisson’s ratio of beams at 3, 7, and 28 days.

Table 10 and Table 11 show the results for UPV tests of the beam with 0.25%, 0.50%, 1.00% 30 mm and 50 mm fibre content. The largest Poisson ratio occurs at 7 days and the highest dynamic elastic modulus occurs at 28 days. The modulus of elasticity is the parameter to determine the material stiffness and it increases with the maturity of concrete. The Poisson’s ratio is determined by the proportion of direct UPV and indirect UPV and as the ratio between direct UPV and indirect UPV increases, the Poisson’s ratio increases. It can also be observed that, according to Table 10 and Table 11, the Poisson’s ratio increases with the decreasing dynamic elastic modulus. Figure 21 shows the dynamic elastic modulus and Figure 22 shows the Poisson’s ratio of beams at 3, 7, and 28 days. The modulus of elasticity increases, and Poisson’s ratio decreases with the increasing fibre content for both 30 mm and 50 mm fibre. The modulus of elasticity is higher for the 50 mm steel FRC than the 30 mm steel FRC in the same steel fibre percentage.

## 5. Recommendations

(1) To obtain a certain relationship between the compressive strength of FRC, the number of experimental cases should be increased. It is recommended to use larger dimensions of specimens to increase the accuracy.
(2) It is recommended to find correlation equations from the statistical analysis of direct, semi-directed, and indirect methods to determine the compressive strength of FRC.
(3) The semi-direct results are low in confidence level. Still, good correlations between semi-direct tests and compressive strength are needed as the semi-direct test could be applicable in most situations.
(4) Different steel fibre lengths and fibre types should be explored in UPV tests as there are various types available and applied in real concrete structures.
(5) To increase the confidence level of UPV to measure the properties of FRC, fibre content could be decided more widely and also matched with real applications. 0.25%, 0.5%, 0.75%, 1% and 1.25% steel fibre content.

## 6. Conclusions

In this paper, empirical results have been developed using the ultrasonic pulse velocity (UPV) test to estimate the values of the modulus of elasticity and Poisson’s ratio of concrete reinforced with steel fibres. The results of the study showed that it is essential to select the setup of the non-destructive test that should be applied (direct, semi-direct, and indirect) since the expected results depend on this setup. It is also necessary to identify the wave types logged by the test equipment because $V_{p}$ or $V_{r}$ velocities must be known for determining the modulus of elasticity or the Poisson’s ratio of fibre-reinforced concrete. However, it is recommended to use the direct test setup whenever possible since this method guarantees the transmission of the maximum pulse energy. The specific findings are presented as follows.

(1) In the long term, the effect of fibre on the rebound hammer result is minimal, while in the short term, the effect is significant. This can confirm that the rebound hammer test is unsuitable for obtaining compressive strength data in FRC early.
(2) The volume fraction of steel fibres seems ineffective in increasing the UPV. However, longer steel fibres can slightly increase the UPV measurements. The UPV in specimens with 50 mm long fibres is larger by 5.7%, 3.8%, and 2.0% at the age of 3, 7, and 28 days, respectively, in comparison to the specimens with 30 mm long fibres. This implies that the fibre content does not affect the measured UPV in matured concrete.
(3) The UPV and rate of strength gain in FRC have a significant difference at an early age of concrete. Both UPV and compressive strength increase when the fibre content increases without changing the fibre length. For the same fibre content, 50 mm FRC shows a higher UPV value and compressive strength than FRC with 30 mm fibre.
(4) The sequence of UPV from largest to smallest is direct UPV, semi-direct UPV, and indirect UPV, respectively. The direct UPV is approximately two times that of indirect UPV. The accuracy of semi-direct UPV is relatively lower.
(5) The ratio between surface velocity and direct velocity can be used to determine the dynamic modulus of elasticity and Poisson’s ratio of the concrete. If the fibre content increases, the dynamic elastic modulus increases, and the Poisson’s ratio decrease for the same length of steel fibre. The modulus of elasticity is higher for the 50 mm steel FRC than the 30 mm steel FRC at the same percentage of steel fibre.

## Figures and Tables

**Table 1:** Properties of steel fibres.

Fibre Material	Length (mm)	Diameter (mm)	Aspect Ratio (l/d)	Specific Gravity	Modulus (GPa)
Steel	30	0.3	100	7.85	200
Steel	50	0.5	100	7.85	200

Note: Aspect ratio: length/diameter.

**Table 2:** Mixture proportion per 1 m^3^.

Quantities	Cement (kg)	Water (kg or L)	Fine Aggregate (kg)	Coarse Aggregate (kg)
Materials (saturated)	487	219	753	816
Materials (dry)	487	253	730	804

**Table 3:** Dimensions and number of specimens.

Specimen Type	Dimensions (mm)	Number
Cube	150×150×150	63
Beam 1	100×100×100	2
Beam 2	100×100×200	2
Beam 3	100×100×300	2
Beam 4	100×100×400	2
Beam 5	100×100×500	7

**Table 4:** Comparative hardness of concrete by rebound hammer test.

Average Rebound	Quality of Concrete
>40	Very good
30–40	Good
20–30	Fair
<20	Poor and/or delaminated
0	Very poor and/or delaminated

**Table 5:** Quality of concrete given by IS code (BS, 1881, 1983) as a function of UPV.

UPV (m/s)	Concrete Quality
>4500	Excellent
3500 to 4500	Good
3000 to 3500	Medium
<3000	Doubtful

**Table 6:** Effect of sample dimensions of pulse transmission (BS 1881: Part 103:1986).

Transducer Frequency (kHz)	Pulse Velocity in Concrete (m/s)		
	V_c_ = 3500	V_c_ = 4000	V_c_ = 4500
	Minimum Permissible Lateral Specimen Dimension (mm)		
24	146	167	188
54	65	74	83
82	43	49	55
150	23	27	30

**Table 7:** Indirect UPV for different path lengths at 3, 7, and 28 days (m/s).

Time (Days)	Fibre Content and Length for 100 mm Path Length						
	0%	0.25% 30 mm	0.50% 30 mm	1.00% 30 mm	0.25% 50 mm	0.50% 50 mm	1.00% 50 mm
3	2192	2610	2736	3014	2962	2959	2240
7	2347	2285	2230	2250	2131	2345	2880
28	2539	2584	2932	4087	3333	3431	3574
	Fibre content and length for 200 mm path length (m/s)						
Time (days)	0%	0.25% 30 mm	0.50% 30 mm	1.00% 30 mm	0.25% 50 mm	0.50% 50 mm	1.00% 50 mm
3	2380	2747	2828	2436	2680	2424	2380
7	1908	2244	2304	1833	1913	2103	2765
28	2754	2906	3164	3498	3909	4073	2987

**Table 8:** Indirect UPV percentage change between 100 mm and 200 mm path length.

Time (Days)	Variation of UPV (m/s)						
	0%	0.25% 30 mm	0.50% 30 mm	1.00% 30 mm	0.25% 50 mm	0.50% 50 mm	1.00% 50 mm
3	8.58	5.25	3.36	−19.18	−9.52	−18.08	6.25
7	−18.7	−1.79	3.32	−18.53	−10.23	−10.32	−3.99
28	8.47	12.46	7.91	−14.41	17.28	18.71	−16.42

**Table 9:** Modulus and Poisson’s ratio for beam without fibre.

Age (Days)	$V_{p}$ (m/s)	$V_{r}$ (m/s)	$V_{p}/V_{r}$	Modulus of Elasticity, $E_{d}$ (GPa)	Poisson’s Ratio, *v*
3	2471	2028	1.218	15.14	0.062
7	3523	2144	1.643	28.10	0.195
28	4574	2558	1.788	45.20	0.229

**Table 10:** Modulus and Poisson’s ratio for beam with 30 mm fibre.

Age (Days)	Fibre Content (%)	$V_{p}$ (m/s)	$V_{r}$ (m/s)	$V_{p}/V_{r}$	Modulus of Elasticity, $E_{d}$ (GPa)	Poisson’s Ratio, v
3	0.25	4194	2380	1.762	38.34	0.223
7		4364	1908	2.287	34.10	0.312
28		4541	2754	1.649	46.59	0.197
3	0.50	4173	2747	1.519	40.79	0.163
7		4370	2244	1.947	39.00	0.260
28		4387	2906	1.510	45.20	0.160
3	1.00	4142	2828	1.465	40.72	0.147
7		4244	2304	1.842	38.20	0.240
28		4504	3164	1.424	48.61	0.134

**Table 11:** Modulus and Poisson’s ratio for beams with 50 mm fibre.

Age (Days)	Fibre Content (%)	$V_{p}$ (m/s)	$V_{r}$ (m/s)	$V_{p}/V_{r}$	Modulus of Elasticity, $E_{d}$ (GPa)	Poisson’s Ratio, v
3	0.25	4355	2436	1.788	40.98	0.229
7		4528	1833	2.470	34.02	0.335
28		4694	3174	1.479	52.14	0.151
3	0.50	4248	2959	1.436	43.13	0.138
7		4393	1913	2.296	34.38	0.314
28		4641	3431	1.353	52.36	0.111
3	1.00	4280	2424	1.766	39.88	0.224
7		4463	2103	2.122	38.05	0.290
28		4642	3573	1.299	52.86	0.092

## Data Availability

The data used to support the findings of this study are included in the article.

## Appendix A

**Table A1:** Aggregate gradation of coarse and fine aggregates.

Sieve Size (mm)	Actual Weight Retained (kg)	Percentage of Weight Retained (%)	Cumulative Percentage Retained (%)	Cumulative Percentage Passing (%)
	Coarse Aggregates			
	0	0	0	100
14	0.005	0.25	0.25	99.75
10	0.315	15.63	15.88	84.12
6.3	0.995	48.88	64.76	35.24
5	0.39	19.35	84.11	15.89
3.35	0.265	13.15	97.26	2.74
2.36	0.04	1.99	99.25	0.75
Pan	0.015	0.7	99.99	0
Fine Aggregates				
2.36	0.22	11	11	89
1.18	0.405	20.25	31.25	68.75
0.6	0.475	23.75	55	45
0.425	0.255	12.75	67.75	32.25
0.3	0.205	10.25	78	22
0.212	0.18	9	87	13
Pan	0.25	12.5	99.5	0

**Table A2:** Concrete mix design for each material [25].

Stage Item		Reference or Calculation	Values	
1	1.1 Characteristic strength	Specified	35 N/mm^2^ at 28 days	
			Proportion defective 5%	
	1.2 Standard deviation	Figure 3	No data 8 N/mm^2^	
	1.3 Margin	C1 or specified	(k = 1.64) 1.64*8 = 13.12 N/mm^2^	
			……. N/mm^2^	
	1.4 Target mean strength	C2	35 + 13.12 = 47.12 N/mm^2^	
	1.5 Cement strength class	specified	42.5	
	1.6 Aggregate type: coarse Aggregate type: fine	Crushed		
		Uncrushed		
	1.7 Free-water/cement ratio	Table 2, Figure 4	0.47	Use the lower values 0.45
	1.8 Maximum free-water/cement ratio	Specified	0.45	
2	2.1 Slump or Vebe time	Specified	Slump 30–180 mm^2^	
	2.2 Maimum aggregate size	Specified	15 mm	
	2.3 Free-water content	Table 3	219 kg/m^3^	
3	3.1 Cement content	C3	219/0.45 = 487 kg/m^3^	
	3.2 Maximum cement content	Specified	……. kg/m^3^	
	3.3 Minimum cement content	Specified	290 kg/m^3^ Use 3.1 if ≤3.2 Use 3.3 if >3.1 422 kg/m^3^	
	3.4 Modified free-water/cement ratio	…………………………………		
4	4.1 Relative density of aggregate (SSD)	2.5 Known		
	4.2 Concrete density	Figure 5	2275 kg/m^3^	
	4.3 Total aggregate content	C4	2275 − 487 − 219 = 1569 kg/m^3^	
5	5.1 Grading of fine aggregate	Percentage passing 600 μm sieve: 45%		
	5.2 Proportion of fine aggregate	Figure 6	48%	
	5.3 Fine aggregate content	C5	1569*0.48 = 753 kg/m^3^	
	5.4 Coarse aggregate content		1569 – 753 = 816 kg/m^3^	
	5.5 Water content	Fine aggregate	(1.85 – 1.795)/1.85 = 3% 22.6 kg water	
		Coarse aggregate	(2.9 – 2.775)/2.9 = 1.4% 11.42 kg water	

Items in italics are optional limiting values that may be specified. Concrete strength is expressed in the units N/mm^2^. 1 N/mm^2^ = 1 MN/m^2^ = 1 MPa. (N = newton; Pa = pascal.) The internationally known term ‘relative density’ used here is synonymous with ‘specific gravity’ and is the ratio of the mass of a given volume of a substance to the mass of an equal volume of water. SSD = based on the saturated surface dry condition.

## References

[1] Sattarifard A.R., Ahmadi M., Dalvand A., Sattarifard A.R. (2022). Fresh and hardened-state properties of hybrid fiber–reinforced high-strength self-compacting cementitious composites. Constr. Build. Mater..

[2] Carrillo J., Ramirez J., Lizarazo-Marriaga J. (2019). Modulus of elasticity and Poisson’s ratio of fiber-reinforced concrete in Colombia from ultrasonic pulse velocities. J. Build. Eng..

[3] Kumar P., Monteiro P. (2014). Concrete Microstructure, Properties and Materials.

[4] Najm H.M., Nanayakkara O., Ahmad M., Sabri Sabri M.M. (2022). Mechanical Properties, Crack Width, and Propagation of Waste Ceramic Concrete Subjected to Elevated Temperatures: A Comprehensive Study. Materials.

[5] Najm H.M., Nanayakkara O., Ahmad M., Sabri Sabri M.M. (2022). Colour Change of Sustainable Concrete Containing Waste Ceramic and Hybrid Fibre: Effect of Temperature. Materials.

[6] Abbass W., Khan M., Mourad S. (2018). Evaluation of mechanical properties of steel fiber reinforced concrete with different strengths of concrete. Constr. Build. Mater..

[7] Parra-Montesinos G. (2005). High-performance fiber-reinforced cement composites: An alternative for seismic design of structures. ACI Struct. J..

[8] Facconi L., Amin A., Minelli F., Plizzari G. (2021). A unified approach for determining the strength of FRC beams subjected to torsion–Part II: Analytical modeling. Struct. Concr..

[9] Hassan A., Jones S. (2012). Non-destructive testing of ultra-high performance fibre reinforced concrete (UHPFRC): A feasibility study for using ultrasonic and resonant frequency testing techniques. Constr. Build. Mater..

[10] Zhang Y., Aslani F., Lehane B. (2021). Compressive strength of rubberized concrete: Regression and GA-BPNN approaches using ultrasonic pulse velocity. Constr. Build. Mater..

[11] Mohammed B.S., Azmi N.J., Abdullahi M. (2011). Evaluation of rubbercrete based on ultrasonic pulse velocity and rebound hammer tests. Constr. Build. Mater..

[12] Hamidian M., Shariati M., Arabnejad M.M.K., Sinaei H. (2011). Assessment of high strength and light weight aggregate concrete properties using ultrasonic pulse velocity technique. Int. J. Phys. Sci..

[13] Saha A.K., Majhi S., Sarker P.K., Mukherjee A., Siddika A., Aslani F., Zhuge Y. (2021). Non-destructive prediction of strength of concrete made by lightweight recycled aggregates and nickel slag. J. Build. Eng..

[14] Kumar S., Rai B. (2019). Pulse velocity–strength and elasticity relationship of high volume fly ash induced self-compacting concrete. J. Struct. Integr. Maint..

[15] Shariq M., Prasad J., Masood A. (2013). Studies in ultrasonic pulse velocity of concrete containing GGBFS. Constr. Build. Mater..

[16] de Oliveira Dias A.R., Amancio F.A., de Carvalho Rafael M.F., Cabral A.E.B. (2018). Study of propagation of ultrasonic pulses in concrete exposed at high temperatures. Procedia Struct. Integr..

[17] Lencis U., Udris A., Korjakins A. (2021). Frost influence on the ultrasonic pulse velocity in concrete at early phases of hydration process. Case Stud. Constr. Mater..

[18] Blitz J., Simpson G. (1996). Ultrasonic Methods of Non-Destructive Testing.

[19] Malhotra V., Carino N. (2004). Non-Destructive Testing of Concrete.

[20] Mohod M.V. (2012). Performance of steel fiber reinforced concrete. Int. J. Eng. Sci..

[21] Saleem M. (2017). Study to detect bond degradation in reinforced concrete beams using ultrasonic pulse velocity test method. Struct. Eng. Mech. Int. J..

[22] Thorpe J. (1988). Simplified Version of The Recommended Practice for Evaluation of Strength Test-Results. Aci. Mater. J..

[23] Haktanır T., Altun F., Karahan O., Arı K., Bekmezci M. (2002). Indirect Determination of In-Situ Compressive Strength of Concrete as a Function of Ultrasonic Pulse Velocity. International Symposium on Structural and Earthquake Engineering 2002.

[24] The Engineering Toolbox. Poisson’s Ratio. The Engineering Toolbox. http://www.engineeringtoolbox.com/poissons-ratio-d_1224.html.

[25] Clayton D., Franklin R.E., Erntroy H.C. (1988). Design of Normal Concrete Mixes.

